# 761. Provider Perceptions of Antimicrobial Use and Educational Needs at Two Community Hospitals Within a Health System

**DOI:** 10.1093/ofid/ofad500.822

**Published:** 2023-11-27

**Authors:** Adam Greenfield, Kathy M Sims, Sangeeta Sastry, Gonzalo Bearman, Michelle Doll, Barry Rittmann

**Affiliations:** VCU Health, Henrico, Virginia; vcu health community memorial hospital, South HIll, Virginia; VCU, Richmond, Virginia; Virginia Commonwealth University Health System, Richmond, VA; Virginia Commonwealth University Health System, Richmond, VA; Virginia Commonwealth University Health System, Richmond, VA

## Abstract

**Background:**

Variability in ASP practices was identified across sites within a recently consolidated health system. A needs assessment was developed to identify knowledge gaps in infectious diseases management and antimicrobial stewardship practices at non-academic, resource-limited community sitesto tailor educational initiatives and promote practice changes.

**Methods:**

A survey was conducted among physicians, pharmacists, advanced practice providers (APPs), and nurses at 2 rural, community hospitals in Virginia containing 80 and 70 beds respectively. The questionnaire contained 6 parts encompassing three domains: confidence in management of infectious diseases, antimicrobial stewardship practices, and educational needs.

**Results:**

The survey was distributed to 134 individuals. A total of 42 respondents completed the survey (response rate 31.3%) including 10 physicians/APPs, 11 pharmacists, 19 nurses, and 2 others.

The most commonly encountered infectious disease syndromes treated in patients is represented in Figure 1. Sepsis and pneumonia were identified as the most common clinical syndromes treated. Overall confidence in managing ID syndromes in accordance with national guidelines was high, particularly among physicians (Figure 2). Pooled results demonstrated greatest confidence in managing urinary tract infection (mean rating 7.8/10) and least confidence in managing organ space/deep surgical site infections (mean 6.7/10).

80% of clinical providersreported evaluating the necessity of antimicrobials dailyand 70% reported utilizing minimal durations of therapy. The most requested topics of education on antimicrobials were: optimization of dosing in special populations, dosing of high-risk medications and strategies for de-escalation. The preferred choice for content delivery was live webinar (36.8%) followed by an online module (31.6%). Newsletter and in-person presentations were less popular (2% each).

**Figure:**
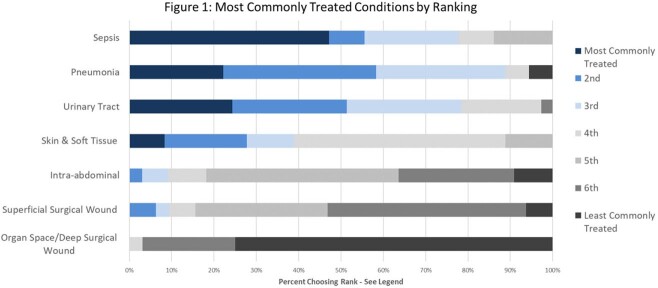

**Figure:**
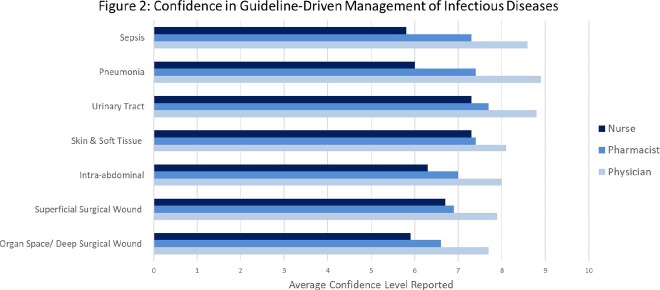

**Conclusion:**

Among healthcare professionals practicing in rural community settings, sepsis was the most common treated syndrome with high confidence in treating all infectious disease syndromes in accordance with guidelines. Future education should focus on both self-reported and demonstrated needs.

**Disclosures:**

**Michelle Doll, MD, MPH**, Molnlycke: Grant/Research Support

